# Causality of Helicobacter pylori infection on eosinophilic esophagitis and potential pathogenesis: a Mendelian randomization study

**DOI:** 10.3389/fimmu.2024.1365604

**Published:** 2024-05-08

**Authors:** Zhenghui Zhu, Yanqing Yang, Xu Han, Lei Peng, Hong Zhu

**Affiliations:** Department of Gastroenterology, The First Affiliated Hospital of Nanjing Medical University, Nanjing, Jiangsu, China

**Keywords:** Helicobacter pylori, eosinophilic esophagitis, inflammatory factors, Mendelian randomization, mediation analysis

## Abstract

**Background:**

Observational studies have indicated a possible connection between Helicobacter pylori (H. pylori) infection and eosinophilic esophagitis (EoE), but their causal relationship has yet to be established. To investigate the causal associations between H. pylori infection and EoE, we performed a Mendelian randomization (MR) analysis.

**Methods:**

Firstly, we conducted both univariable and multivariable Mendelian randomization (MR) analyses. Furthermore, a two-step MR was carried out to ascertain the potential underlying pathways of these associations, particularly the involvement of inflammatory cytokines. We employed the inverse-variance weighted (IVW) method as the main analysis in our MR study. To enhance the credibility of the results, we also conducted several sensitivity analyses.

**Results:**

Our study demonstrated a noteworthy correlation between genetically predicted anti-H. pylori IgG antibody levels and a reduced risk of EoE (OR=0.325, 95% CI=0.165–0.643, P value=0.004, adj p value=0.009). No significant causal associations were detected between other H. pylori antibodies and EoE in our study. When it comes to multivariable MR analysis controlling for education attainment, household income, and deprivation individually, the independent causal impact of anti-H. pylori IgG on EoE persisted. Surprisingly, the two-step MR analysis indicated that inflammatory factors (IL-4, IL-5, IL-13, IL-17, and IFN-γ) did not appear to mediate the protective effect of H. pylori infection against EoE.

**Conclusion:**

Findings suggested that among the range of H. pylori-related antibodies, anti-H. pylori IgG antibody is the sole causal factor associated with protection against EoE. Certain inflammatory factors may not be involved in mediating this association. These findings make a significant contribution to advancing our understanding of the pathogenesis of EoE and its evolving etiology.

## Introduction

Eosinophilic esophagitis (EoE) is an inflammatory disease characterized by signs of esophageal dysfunction, such as difficulty in swallowing or food blockage (1, 2). It was defined by the presence of a significant degree of mucosal eosinophilic infiltration exceeding 15 eosinophils per high-power field in the esophagus (3). Currently, EoE has developed from an infrequently reported disorder to a prevalent condition in clinical settings. Recent evidence strongly suggests that changes in the esophageal microbiota may have a significant impact on the development of esophageal inflammation in EoE (4).

Helicobacter pylori (H. pylori) is a microaerophilic bacterium known to be responsible for various gastrointestinal conditions, including gastritis, peptic ulcers, and neoplastic diseases (5, 6). In recent years, a number of observational studies have suggested an inverse association between H. pylori infection rates and EoE prevalence (7, 8). A meta-analysis reported that H. pylori infection was linked to a reduced risk of EoE in a large sample size (N= 377,795) (9). However, no evidence of a protective effect of H. pylori against EoE was found in a prospective study by Molina-Infante et al (10). Thus, the relationship and underlying mechanisms between H. pylori infection and EoE needs to be further clarified. According to previous research, several inflammatory mediators, including interleukin-4 (IL-4), interleukin-5 (IL-5), and interleukin-13 (IL-13) mediate tissue inflammation and regulate extracellular matrix deposition in EoE (11). In addition, high interferon-γ (IFN-γ) significantly increases EoE risk and engages in the progress of EoE (12). Furthermore, H. pylori infection is thought to be a risk factor for inflammation (13, 14). Thus, we assumed that inflammatory factors might serve as mediators for the influence of H. pylori infection on EoE. Additionally, some potential confounding factors such as socioeconomic status may affect the true associations between H. pylori and EoE (15).

In this study, we initially conducted univariable Mendelian randomization (MR) analysis and subsequently multivariable MR analysis with the genome-wide association studies (GWAS) datasets to examine causality between H. pylori infection and EoE (16, 17). Furthermore, a two-step MR analysis was carried out to delve deeper into the underlying mechanisms.

## Methods

### Study design and data source

**Figure 1:**
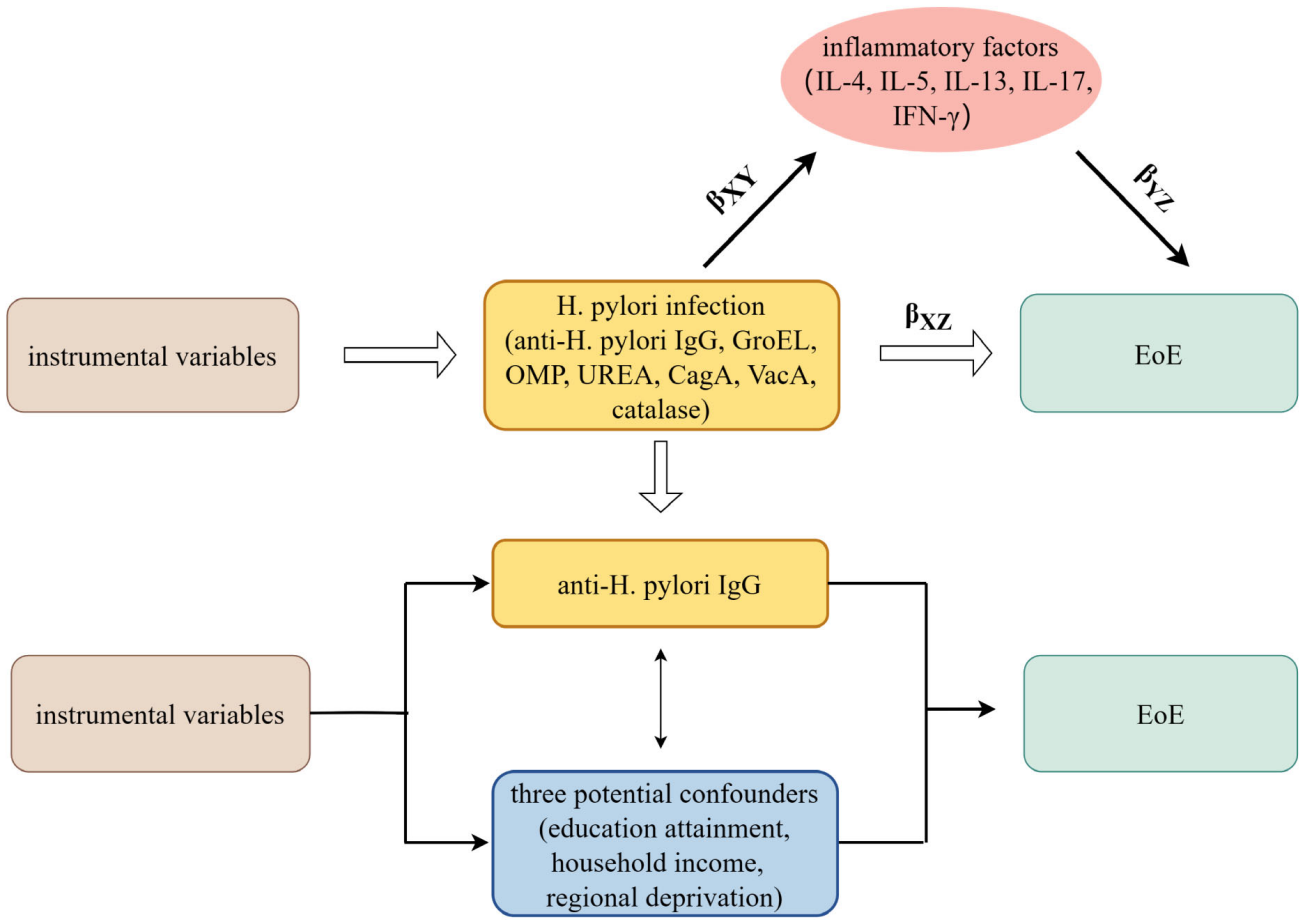
The overview design in this MR study. MR and Multivariate MR analyses investigate the effects of seven H. pylori antibodies on EoE; Two-step MR analysis evaluates the roles of inflammatory factors mediating the association between IgG-positive H. pylori infection and EoE. β_XZ_, the overall impact of anti-H. pylori IgG antibody on EoE; β_XY_β_YZ_, the mediated effect, or indirect effect, of anti-H. pylori IgG antibody on EoE via inflammatory factors; H. pylori, Helicobacter pylori; EoE, eosinophilic esophagitis; GroEL, chaperonin GroEL; OMP, outer membrane protein; UreA, urease subunit-A; VacA, vacuolating cytotoxin-A; CagA, cytotoxin-associated gene-A. MR, Mendelian randomization.

A brief description of this MR design is shown in **Figure 1**. Using GWAS summary statistics, we conducted univariable and multivariable two-sample MR analyses to investigate the possible causal impact of H. pylori infection on the development of eosinophilic esophagitis (EoE). Additionally, a two-step MR study was carried out to examine the potential role of inflammatory factors as mediators between the effect of H. pylori infection and EoE. H. pylori infection was determined by detecting serum-specific antibodies targeting the H. pylori protein. Patients with H. pylori infection exhibited the presence of antibodies such as anti-H. pylori IgG, chaperonin GroEL (GroEL), outer membrane protein (OMP), urease subunit-A (UreA), vacuolating cytotoxin-A (VacA), cytotoxin-associated gene-A (CagA), and catalase (18). Summary-level data for seven H. pylori antibodies was obtained from GWAS datasets comprising a cohort of 8,735 individuals in the UK Biobank.

As to EoE, we extracted the summary statistics from the extensive GWAS meta-analyses conducted by Chang et al (19). The GWAS on EoE by Chang et al. included data from four independent cohorts (CHOP-1, CHOP-2, CHOP-3, and eMERGE) with a total of 15,564 subjects (1,930 cases and 13,634 controls matched for ancestry). The sample was predominantly composed of individuals with White European descent. A detailed description of the study cohorts and methods used can be found in Chang et al. Summary statistics for IL-4, IL-5, IL-13, IL-17 and IFN-γ were obtained from the Cardiovascular Risk in Young Finns (YFS) and FINRISK GWAS (20). Socioeconomic status was assessed through various indicators, including educational attainment (measured by age at completion of full-time education), household income (calculated as the average total household income before tax), and regional deprivation (measured using the Townsend deprivation index) (21). Summary statistics of socioeconomic status were downloaded from corresponding release GWASs from the UK Biobank (22, 23). The GWASs included in our study are listed in **Supplementary Table 1**.

### Selection of the genetic instruments

In line with previous MR studies, we adopted a less stringent criterion (p<5×10^−6^) to identify a broader range of SNPs associated with H. pylori infection, socioeconomic status, and inflammatory factors in our current investigation (24, 25). To ensure the independence of SNPs, we employed a linkage disequilibrium (LD) clumping algorithm (r^2^<0.001 within 10,000 kb), effectively removing SNPs that exhibited strong LD. R^2^ values represents the amount of variance in exposure actors that could be attributed to the instrumental variables. Additionally, we calculated F-statistics to assess the strength of the selected instrumental variables (26).

### Mendelian randomization

Initially, a univariable MR analysis was conducted to examine the causal effects of H. pylori infection on EoE. The primary analysis in our study involved the utilization of the inverse-variance weighted (IVW) method (27, 28). In instances where heterogeneity was detected, assessed by Cochran’s Q test, we applied a random effects model (29). Moreover, in order to enhance the reliability of the findings, we performed several supplementary analyses such as MR-Egger and weighted median. MR-Egger regression allows detection and correction for directed pleiotropy, which occurs when genetic instruments influence outcome through pathways other than exposure. Weighted median analysis corrects for the effect of invalid instrumental variables (IVs) with robust estimates even in situations with 50% invalid IVs making it a valuable tool. Consistency in the direction of results across different approaches is considered as sufficient evidence to claim a causal effect, thereby enhancing the robustness of the study findings (17). Then, we adopt multivariable MR analysis, an extension of univariable MR that allows for the estimation of causal effects from multiple risk factors simultaneously (30, 31). To investigate the potential mediation of inflammatory factors on EoE, we conducted a two-step MR study. The overall impact of H. pylori infection on EoE is equal to β_XZ_. The mediated effect, or indirect effect, of H. pylori infection on EoE via inflammatory factors is equal to β_XY_β_YZ_. The direct impact of H. pylori infection on EoE is given by β_XZ_ - β_XY_β_YZ_ (32, 33). **Supplementary Table S2** includes a glossary of terms frequently utilized in MR studies. To account for multiple tests, we applied the Benjamini-Hochberg correction to adjust the p-values (p<0.05), which helps control the false discovery rate. All statistical analyses were two-sided and conducted using the “TwoSampleMR” and “MendelianRandomization” packages in R software.

### Sensitivity analysis

Sensitivity analysis was performed to ensure robustness of results. We examined the p-value of the MR-Egger intercept to evaluate the potential impact of pleiotropy, as it can introduce bias into the MR findings (34). To account for pleiotropy and identify potential outliers, we employed the MR-PRESSO method before proceeding with MR estimates (35). Heterogeneity was detected through Cochran’s Q test, indicating a p-value of less than 0.05.

## Results

### Causal effect of Helicobacter pylori infection on eosinophilic esophagitis

**Figure 2:**
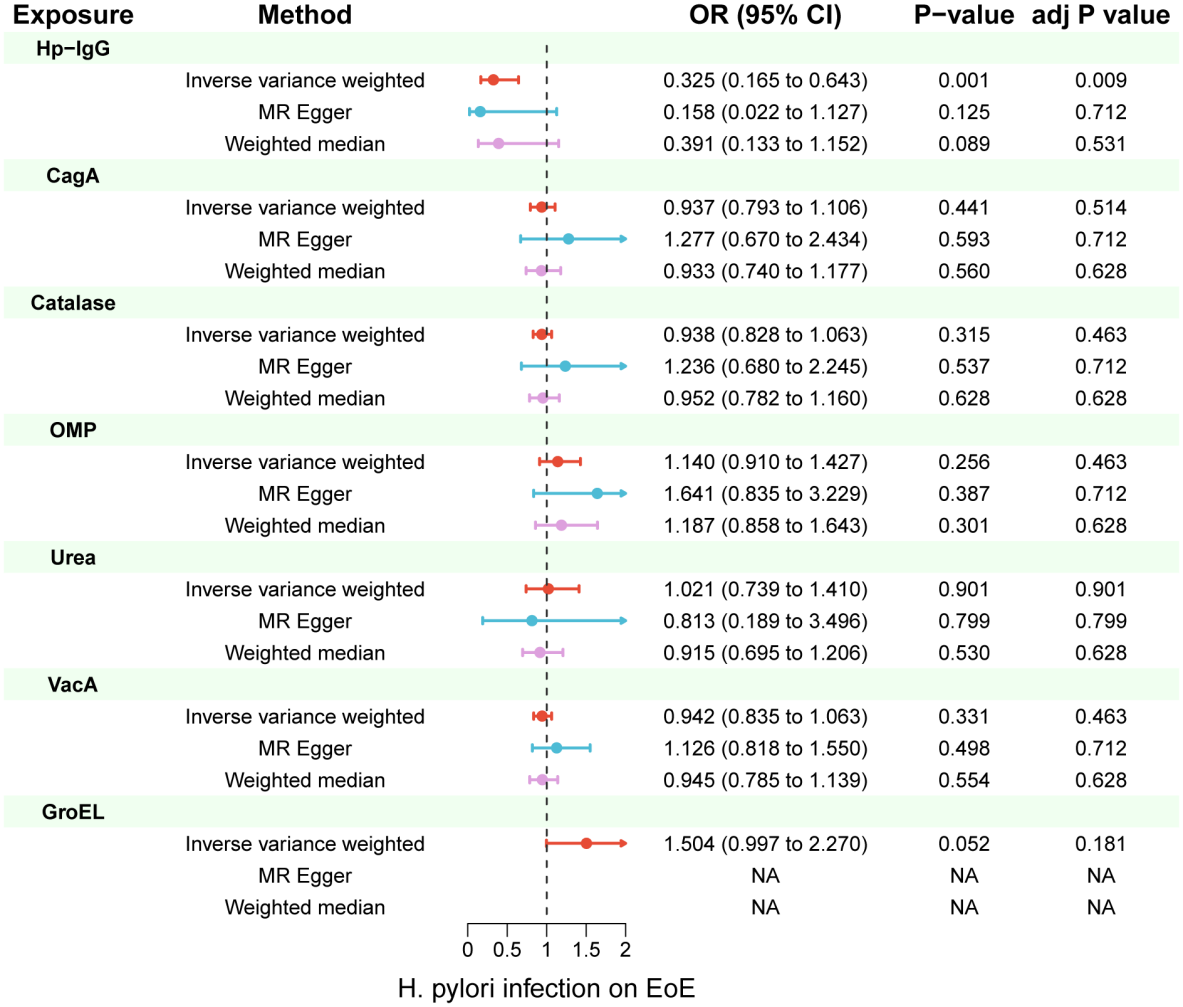
Inverse-variance weighted (IVW), MR-Egger and weighted median from all the primary MR analyses are shown for the effects of seven H. pylori antibodies on EoE. IVW adj p Value< 0.05 (Benjamini-Hochberg correction for multiple testing) coupled with directionally consistent results of MR-Egger and weighted median analyses was considered as sufficient evidence to claim a causal effect. MR, Mendelian randomization.

Due to ambiguous palindrome, we removed one SNP for antibody levels of CagA, GroEL, OMP, and VacA respectively. In the final MR analysis, we identified 7, 3, 5, 2, 3, 5, and 7 SNPs closely related to anti-H. pylori IgG, GroEL, OMP, UREA, CagA, VacA, and catalase respectively (**Supplementary Table S3**). All genetic instruments employed in the MR analysis have an F-statistic exceeding 10 (**Supplementary Table S4**). Higher levels of anti-H. pylori IgG antibodies, as determined by genetic predictions, were associated with a lower risk of EoE (OR=0.325, 95% CI=0.165–0.643, P value=0.004) in the IVW model (**Figure 2**). The association persisted after correction for multiple testing (adj p value=0.009). While not reaching statistical significance, both the MR-Egger (OR=0.158, 95% CI=0.022–1.127, P value=0.125, adj p value=0.712) and weighted median (OR=0.391, 95% CI=0.133–1.152, P value=0.089, adj p value=0.531) results still indicated a protective effect of anti-H. pylori IgG against EoE. The leave-one-out method showed that the results remained reliable after removing any one of the SNPs except for rs111575036 (**Supplementary Figure S1**). Conversely, no significant causal associations were observed between CagA, Catalase, GroEL, OMP, Urea, VacA, and EoE (**Table 1**). There is no evidence of potential horizontal pleiotropy in either the MR-PRESSO Global or the MR Egger Intercept tests (all p values > 0.05) (**Table 2**). Furthermore, Cochran’s Q test did not show any statistical evidence of heterogeneity except for the Urea antibody (Q=10.778, P value=0.029). Nevertheless, no indication of heterogeneity was found in the funnel plot (**Supplementary Figure S2**).

**Table 1:** Univariable Mendelian randomization using IVW, MR-Egger and weighted median analyses estimates the effects of seven H. pylori antibodies on EoE.

exposure	method	nSNP	OR (95% CI)	P-value	adj P value
Hp-IgG					
	IVW	7	0.325 (0.165 to 0.643)	0.001	0.009
	MR Egger	7	0.158 (0.022 to 1.127)	0.125	0.712
	Weighted median	7	0.391 (0.133 to 1.152)	0.089	0.531
CagA					
	IVW	3	0.937 (0.793 to 1.106)	0.441	0.514
	MR Egger	3	1.277 (0.670 to 2.434)	0.593	0.712
	Weighted median	3	0.933 (0.740 to 1.177)	0.56	0.628
Catalase					
	IVW	5	0.938 (0.828 to 1.063)	0.315	0.463
	MR Egger	5	1.236 (0.680 to 2.245)	0.537	0.712
	Weighted median	5	0.952 (0.782 to 1.160)	0.628	0.628
OMP					
	IVW	3	1.140 (0.910 to 1.427)	0.256	0.463
	MR Egger	3	1.641 (0.835 to 3.229)	0.387	0.712
	Weighted median	3	1.187 (0.858 to 1.643)	0.301	0.628
Urea					
	IVW	5	1.021 (0.739 to 1.410)	0.901	0.901
	MR Egger	5	0.813 (0.189 to 3.496)	0.799	0.799
	Weighted median	5	0.915 (0.695 to 1.206)	0.53	0.628
VacA					
	IVW	7	0.942 (0.835 to 1.063)	0.331	0.463
	MR Egger	7	1.126 (0.818 to 1.550)	0.498	0.712
	Weighted median	7	0.945 (0.785 to 1.139)	0.554	0.628
GroEL					
	IVW	2	1.504 (0.997 to 2.270)	0.052	0.181

*nSNP, number of single nucleotide polymorphism; IVW, inverse variance weighted; GroEL, chaperonin GroEL; OMP, outer membrane protein; UreA, urease subunit-A; VacA, vacuolating cytotoxin-A; CagA, cytotoxin-associated gene-A; EoE, eosinophilic esophagitis.

**Table 2:** Sensitivity analysis of seven H. pylori antibodies on risk of EoE.

exposure	method	Cochran Q p value	Egger intercept	P-Egger intercept	MR-PRESSO P-value
Hp-IgG					0.542
	IVW	0.57			
	MR Egger	0.523	0.05	0.47	
CagA					——
	IVW	0.487			
	MR Egger	0.497	-0.118	0.503	
**Catalase**					0.638
	IVW	0.609			
	MR Egger	0.61	-0.179	0.418	
OMP					——
	IVW	0.512			
	MR Egger	0.984	-0.08	0.454	
Urea					0.056
	IVW	0.029			
	MR Egger	0.015	0.067	0.773	
VacA					0.696
	IVW	0.679			
	MR Egger	0.783	-0.069	0.271	
GroEL					——
	IVW	0.237	——	——	

Cochran-Q test is used to analyze whether there was heterogeneity, and IVW p<0.05 indicated heterogeneity. MR-Egger and MR-PRESSO analyses are used to analyze whether there was pleiotropy. MR-Egger intercept p-value or MR-PRESSO p-value <0.05 indicated pleiotropy.

*IVW, inverse variance weighted; GroEL, chaperonin GroEL; OMP, outer membrane protein; UreA, urease subunit-A; VacA, vacuolating cytotoxin-A; CagA, cytotoxin-associated gene-A.

**Figure 3:**
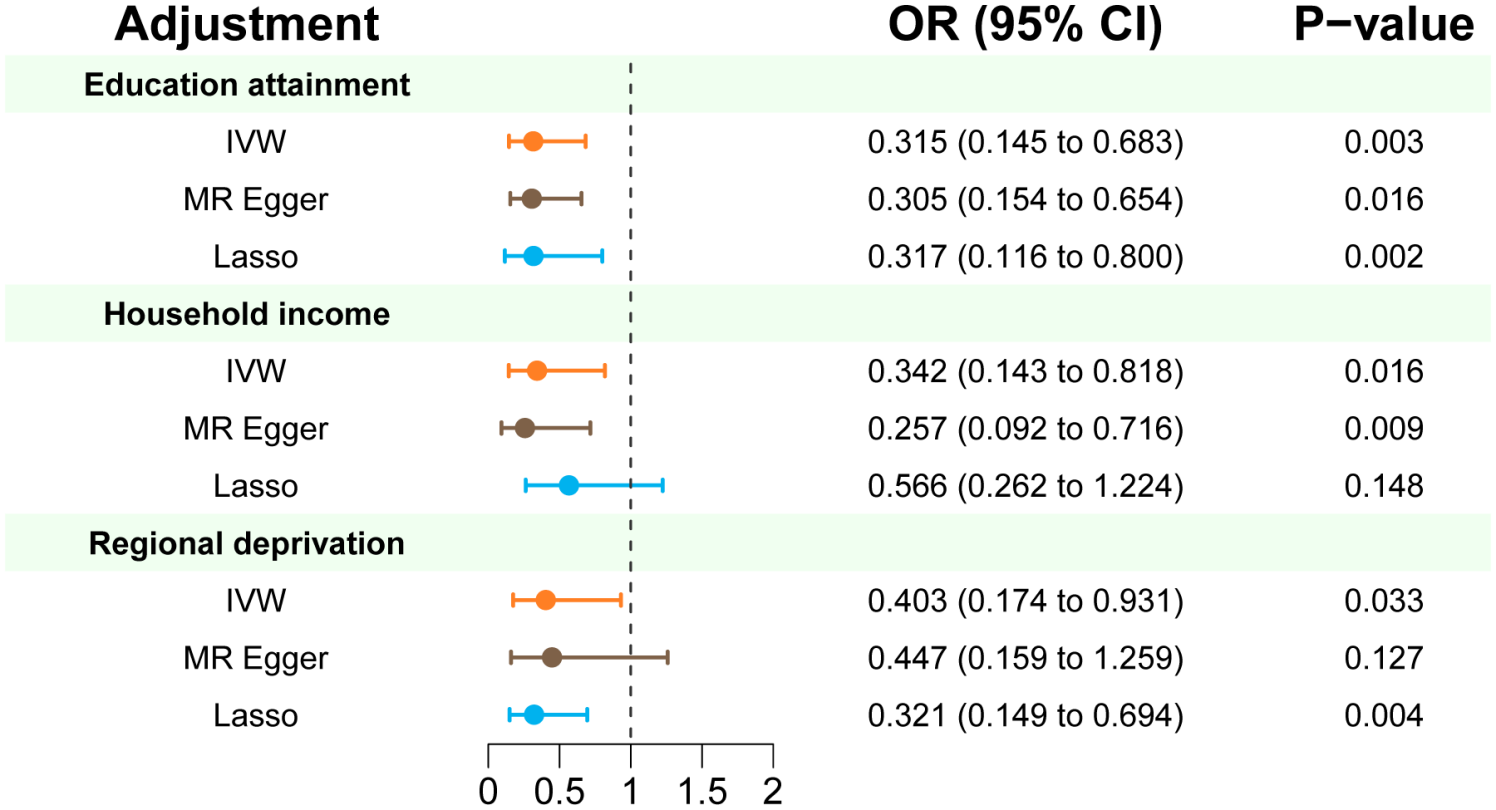
Multivariate MR estimates of the effect of anti-H. pylori IgG antibody on risk of EoE adjusted for education attainment, household income, and deprivation individually. IVW p < 0.05 coupled with directionally consistent results of MR-Egger and Lasso analyses was considered as sufficient evidence to claim a causal effect.

In multivariable MR analysis controlling education attainment, household income, and deprivation individually, the independent causal effect of anti-H. pylori IgG on EoE remained (controlling for education attainment: OR=0.315, P value=0.003; controlling for household income: OR=0.342, P value=0.016; controlling for deprivation: OR=0.403, P value=0.033) (**Figure 3**). Consistent findings were observed across different analytical methods (**Table 3**). Moreover, the results of multivariable MR-Egger intercept test (all p values > 0.05) supports the reliability of the findings.

**Table 3:** Multivariate MR analysis of anti-H. pylori IgG antibody on the risk of EoE after adjustment of education attainment, household income, and deprivation individually.

Adjustment	Method	nSNP	OR (95% CI)	P-value	Cochran Q p value	P-Egger intercept
Education attainment						
	IVW	74	0.315 (0.145 to 0.683)	0.003	0.185	
	Egger	74	0.305 (0.154 to 0.654)	0.016		0.915
	Lasso	74	0.317 (0.116 to 0.800)	0.002		
Household income						
	IVW	79	0.342 (0.143 to 0.818)	0.016	0.001	
	Egger	79	0.257 (0.092 to 0.716)	0.009		0.298
	Lasso	79	0.566 (0.262 to 1.224)	0.148		
Regional deprivation						
	IVW	62	0.403 (0.174 to 0.931)	0.033	0.086	
	Egger	62	0.447 (0.159 to 1.259)	0.127		0.736
	Lasso	62	0.321 (0.149 to 0.694)	0.004		

*nSNP, number of single nucleotide polymorphism; IVW, inverse variance weighted.

### Mediation analysis

**Figure 4:**
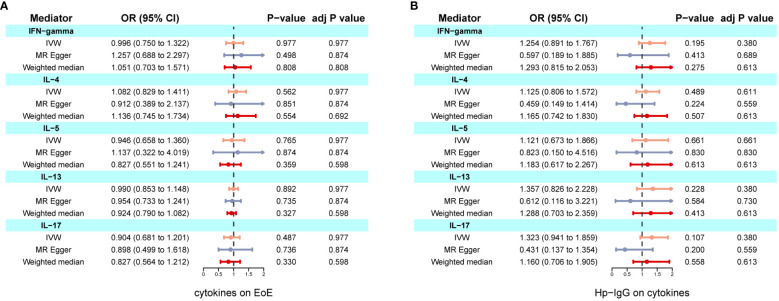
Two-step MR investigating IL-4, IL-5, IL-10, IL-13, and IFN-γ as mediators of the effect of anti-H. pylori IgG antibody on EoE. **(A)** Causal effects of inflammatory factors on EoE; **(B)** Causal effects of anti-H. pylori IgG antibody on inflammatory factors. IVW adj p Value< 0.05 (Benjamini-Hochberg correction for multiple testing) coupled with directionally consistent results MR-Egger and weighted median analyses was considered as sufficient evidence to claim a causal effect.

We found evidence of pleiotropy (global test P-value = 0.024) between IFN-γ and EoE and identified one possible pleiotropic SNP (rs112783231) using the MR-PRESSO analysis. All genetic instruments of inflammatory factors were suitable for MR analysis with an F-statistic greater than 10 (**Supplementary Table S5**). After removing the SNP, pleiotropy and heterogeneity were absent (**Supplementary Table S6**). Besides, there is no evidence of causal effects between inflammatory factors and EoE (**Figure 4A**). Similarly, anti-H. pylori IgG was not significantly related to all inflammatory factors including IL-4, IL-5, IL-10, IL-13, and IFN-γ. The results from all methods used are presented in **Figure 4B**. There was no statistical evidence of pleiotropy between anti-H. pylori IgG and all inflammatory factors (**Supplementary Table S7**). In conclusion, there is no evidence that inflammatory factors mediate the causation of H. pylori infection and EoE.

## Discussion

In the current study, we explored possible causal links of H. pylori infection and EoE risk. Our results showed that anti-H. pylori IgG antibody levels were related to EoE. Remarkably, the inverse causal effects remained consistent and stable in both univariable MR and multivariable MR analyses. Furthermore, the findings from mediation analysis indicated that inflammatory factors may not act as the mediator.

Existing evidence for the association between H. pylori infection and EoE comes from observational studies. However, it has not been possible to draw confident causal conclusions due to confusion bias and reverse causality. In alignment with our results, a large cross-sectional study from the United States pathology database of 165,017 patients found that patients with EoE had reduced odds of H. pylori infection (36). H. pylori infection was also shown to be an independent protective factor for EoE in another case-control study of 966 histological biopsy samples (37). Furtherly, in a recent study comprising of 596,479 individuals, there is a strong inverse association of H. pylori and EoE (38). Our findings using an MR approach effectively address confounding issues that are inherent in observational analyses and suggest a causal relationship between H. pylori infection and EoE.

Within the framework of the “hygiene hypothesis”, lower socioeconomic and hygienic conditions may contribute to the increased incidence of H. pylori infection and decreased allergy-related disease in developing countries. Given that EoE is considered an allergic disease, some studies have proposed that H. pylori may only be a surrogate for these factors rather than a protector against atopic disease (10). Thus, we performed multivariable MR to investigate whether socioeconomic environment may act as a potential confounder for this association. More compelling, genetic liability to H. pylori infection remained linked to EoE, after adjustment for separate effects of anti-H. pylori antibody levels on education attainment, household income, and deprivation.

The underlying mechanism explaining how H. pylori infection may decrease EoE risk remains to be elucidated. However, experimental evidence confirms that H. pylori displays a beneficial immunoregulatory effect, which also play a role in conditions including asthma and allergies through modifying signaling pathways (39–41). H. pylori infection triggers the expression of IFN-γ and IL-17, stimulating Th1 and Th17 cells while concurrently suppressing Th2 cells linked to allergic responses (42–44). Additionally, it is well-established that EoE is an allergic condition distinguished by a Th2 immune response, which manifests as an excessive release of inflammatory cytokines such as IL-4, IL-5, and IL-13. Thus, it is hypothesized that H. pylori infection could potentially protect against EoE through an imbalanced Th1/Th2 system, and causative relationships may be mediated by inflammatory factors. However, our mediation analysis findings do not support this hypothesis, indicating that the causal connection of H. pylori infection and EoE may be influenced by alternative inflammatory factors or pathways. Several possible explanations could shed light on the causal role of H. pylori infection on EoE. Infection of the stomach by H. pylori induces alterations in the upper gastrointestinal microbiome, which may impact the esophagus and its vulnerability to the development of eosinophilic esophagitis (45). Furthermore, chronic H. pylori infection can lead to gastritis, resulting in reduced gastric acid secretion, which may provide partial protection against developing EoE (46, 47).

This study represents the first MR analysis of the causal association between H. pylori infection and EoE and their possible pathogenesis. Our findings successfully overcome the inherent limitations of observational studies. However, it is important to consider several limitations that were present in our research. Firstly, we adopted a more lenient threshold (p<5×10^-6^) when selecting instrumental variables, which may introduce a weak instrument bias into the overall estimates. Secondly, the potential residual pleiotropy is challenging to exclude, even though multiple analyses have been conducted to assess pleiotropy. Additionally, the results of multivariable Mendelian randomization suggest that there is still a causal relationship between H. pylori infection and EoE, but this cannot be construed as evidence that other confounding is absent (48). Thirdly, leave-one-out analysis showed that the association between H. pylori infection and EoE may be partially mediated through other causal pathways related to rs111575036. We queried the PhenoScanner website (http://www.phenoscanner.medschl.cam.ac.uk/) and found that rs111575036 was associated with Parkinson’s disease and cg25815185 methylation (p<5×10^−6) (23, 49). However, there is no any literature indicating a mechanistic link between Parkinson’s disease, cg25815185 methylation, and the pathogenesis of EoE or its related characteristics. Further research is needed to elucidate the functional significance of rs111575036 and its association with the observed effects. Finally, it is crucial to acknowledge that H. pylori antibodies may not reflect current or persistent infection status (50). A potential protective role for H. pylori could exist through early colonization, which may eventually protect against the development of further allergic disorders. It is also plausible that associated immune responses resulting from an existing infection might contribute to this relationship. Our research provides novel perspectives and insight to the potential pathways that could explain the association between anti-H. pylori IgG and EoE.

## Conclusion

In conclusion, our study contributes to the expanding body of evidence that supports a robust inverse association between H. pylori infection and EoE. This finding holds significant implications for future research aimed at understanding the pathogenic role of H. pylori in EoE.

## Data availability statement

The original contributions presented in the study are included in the article/**Supplementary Material**. Further inquiries can be directed to the corresponding authors.

## Ethics statement

Ethical approval was not required for the study involving humans in accordance with the local legislation and institutional requirements. Written informed consent to participate in this study was not required from the participants or the participants’ legal guardians/next of kin in accordance with the national legislation and the institutional requirements.

## Author contributions

ZZ: Formal Analysis, Methodology, Software, Validation, Visualization, Writing – original draft, Writing – review & editing. YY: Validation, Writing – original draft. XH: Validation, Writing – original draft. LP: Conceptualization, Formal Analysis, Funding acquisition, Project administration, Software, Visualization, Writing – review & editing. HZ: Conceptualization, Data curation, Funding acquisition, Investigation, Methodology, Project administration, Resources, Supervision, Visualization, Writing – review & editing.
