# Supplementary material for: Causality of Helicobacter pylori infection on eosinophilic esophagitis and potential pathogenesis: a Mendelian randomization study
Source: Front Immunol. 2024 May 8;15:1365604. doi: 10.3389/fimmu.2024.1365604 (PMC11109363; doi:10.3389/fimmu.2024.1365604)
Supplement: Supplementary file 1 [file DataSheet_1.zip › supplementary material/supplementary material.docx]

**Causality of Helicobacter pylori infection on eosinophilic esophagitis and potential pathogenesis: a Mendelian randomization study**

Zhenghui Zhu^1^ Yanqing Yang^1^ Xu Han^1^ Lei Peng^1*^ Hong Zhu^1*^

^1^Department of Gastroenterology, The First Affiliated Hospital of Nanjing Medical University, Nanjing, Jiangsu, China

*Correspondence to:

Lei Peng. Department of Gastroenterology, First Affiliated Hospital of Nanjing Medical University, Nanjing, China. Email: penglei@njmu.edu.cn

Hong Zhu. Department of Gastroenterology, First Affiliated Hospital of Nanjing Medical University, Nanjing, China. Email: zhuhong1059@126.com

**Supplementary material**

**Supplementary Table S1.** Summary of genome-wide association studies (GWAS) datasets in our study.

| **Phenotype** | **Consortium/Author** | **Year** | **Sample size (N)** | **SNPs (N)** | **PMID** | **OpenGWAS ID** |
| --- | --- | --- | --- | --- | --- | --- |
| **Exposure** |  |  |  |  |  |  |
| Anti-H. pylori IgG | Butler-Laporte et al | 2020 | 8,735 | 7,646,298 | 33204752 | ebi-a-GCST90006910 |
| CagA | Butler-Laporte et al | 2020 | 985 | 9,165,056 | 33204752 | ebi-a-GCST90006911 |
| Catalase | Butler-Laporte et al | 2020 | 1,558 | 9,167,570 | 33204752 | ebi-a-GCST90006912 |
| GroEL | Butler-Laporte et al | 2020 | 2,716 | 9,172,299 | 33204752 | ebi-a-GCST90006913 |
| OMP | Butler-Laporte et al | 2020 | 2,640 | 9,167,440 | 33204752 | ebi-a-GCST90006914 |
| UreA | Butler-Laporte et al | 2020 | 2,251 | 9,170,248 | 33204752 | ebi-a-GCST90006915 |
| VacA | Butler-Laporte et al | 2020 | 1,571 | 9,178,635 | 33204752 | ebi-a-GCST90006916 |
| **Outcome** |  |  |  |  |  |  |
| EoE* | Chang X et al | 2021 | 15,564 | - | [34506852](https://www.ebi.ac.uk/gwas/publications/34506852) | GCST90027899 |
| **Adjustment** |  |  |  |  |  |  |
| Educational attainment | MRC-IEU | 2018 | 307,897 | 9,851,867 | - | ukb-b-6134 |
| household income | MRC-IEU | 2018 | 397,751 | 9,851,867 | - | ukb-b-7408 |
| regional deprivation | MRC-IEU | 2018 | 462,464 | 9,851,867 | - | ukb-b-10011 |
| **Mediator** |  |  |  |  |  |  |
| interleukin-4 | Ahola-Olli et al | 2016 | 8,124 | 9,786,064 | 27989323 | ebi-a-GCST004453 |
| interleukin-5 | Ahola-Olli et al | 2016 | 3,364 | 9,450,731 | 27989323 | ebi-a-GCST004452 |
| interleukin-13 | Ahola-Olli et al | 2016 | 3,557 | 9,539,073 | 27989323 | ebi-a-GCST004443 |
| interleukin-17 | Ahola-Olli et al | 2016 | 7,760 | 9,786,653 | 27989323 | ebi-a-GCST004442 |
| interferon-γ | Ahola-Olli et al | 2016 | 7,701 | 9,785,363 | 27989323 | ebi-a-GCST004456 |

SNPs, single nucleotide polymorphisms; N, number; GroEL, chaperonin GroEL; OMP, outer membrane protein; UreA, urease subunit-A; VacA, vacuolating cytotoxin-A; CagA, cytotoxin-associated gene-A; EoE, eosinophilic esophagitis. *: [GWAS Catalog (ebi.ac.uk)](https://www.ebi.ac.uk/gwas/downloads/summary-statistics).

**Supplementary Table S2.** Glossary of terms commonly used in MR studies.

| Genome-Wide Association Studies (GWAS) are large-scale studies that assess genetic variations across the entire genome to identify genetic factors associated with complex traits, diseases, or phenotypes. |
| --- |
| Summary statistics refer to aggregate numerical results that summarize the associations between genetic variants across the entire genome and a particular trait or disease. These summary statistics are derived from analyzing data collected in a GWAS, where researchers investigate millions of genetic markers (single nucleotide polymorphisms, SNPs) in individuals to identify variants associated with specific traits or diseases. |
| An SNP is a single nucleotide variation resulting from a divergence in one DNA base. |
| Mendelian randomization (MR) utilizes genetic variants as instruments to estimate causal effects of risk factors on disease outcomes. The random allocation of genetic variants during meiosis mimics a natural randomized experiment, reducing concerns about confounding commonly encountered in observational studies. |
| Linkage disequilibrium (LD) refers to the non-random association of alleles at different loci, with frequencies exceeding those expected based on their individual frequencies. This phenomenon is particularly prevalent among genetic variants in close physical proximity and can be leveraged for gene mapping purposes. |
| Mediator variable provides a link between exposure and outcome; also known as intermediate phenotype. |


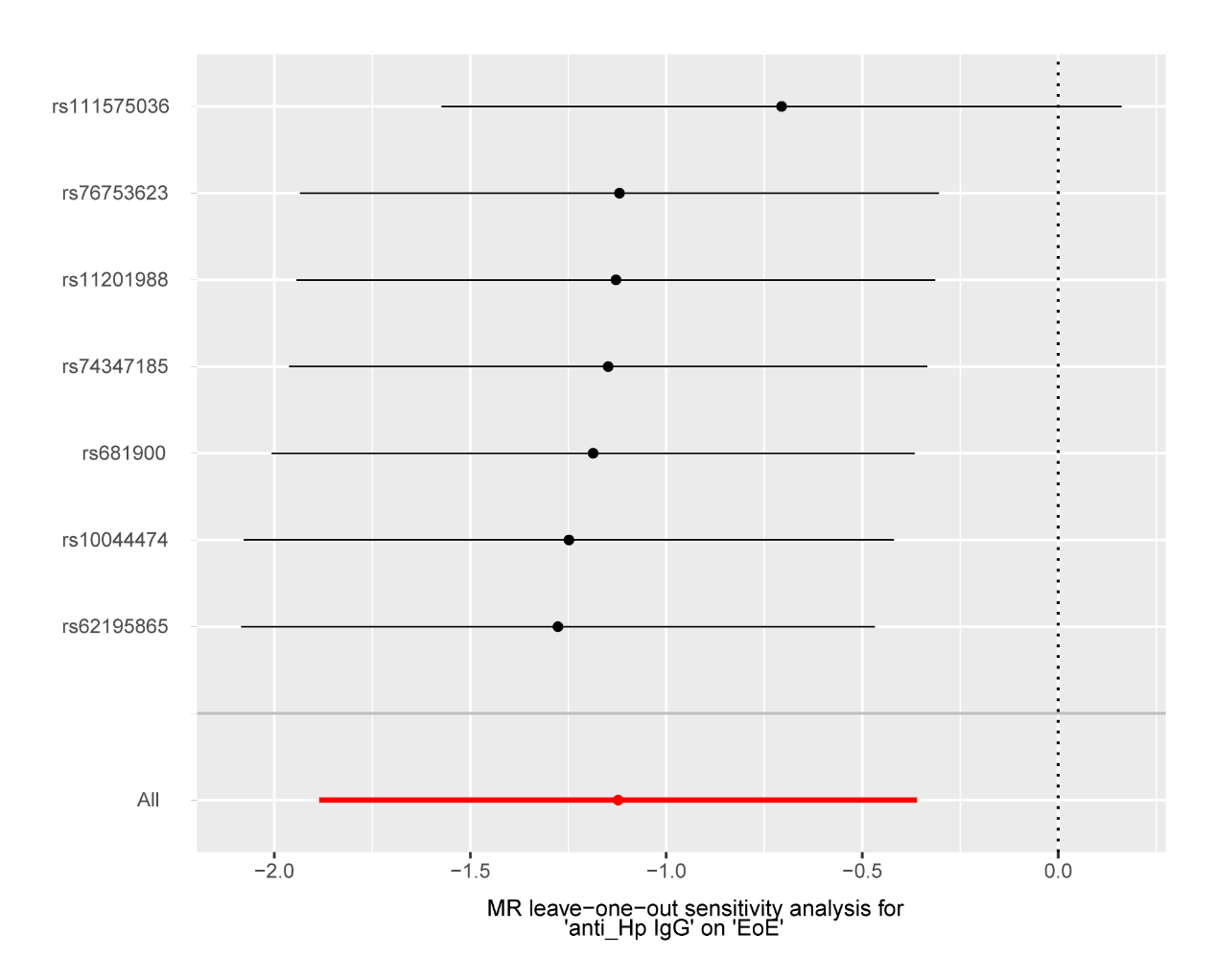


**Supplementary** **Figure 1.** The leave-one-out sensitivity analysis of the association between anti-H. pylori IgG antibody on EoE in MR analysis. EoE, eosinophilic esophagitis.


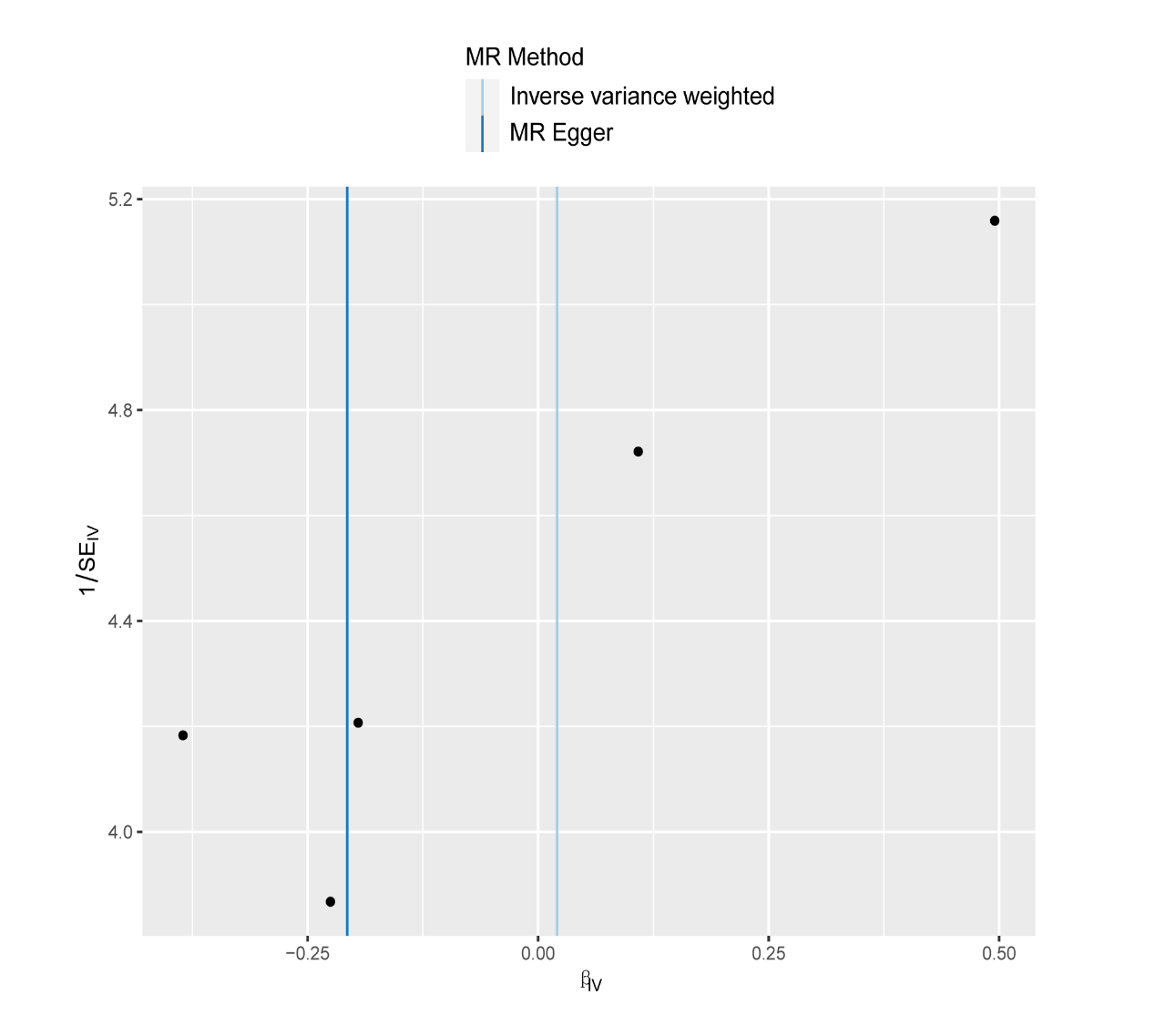


**Supplementary** **Figure 2.** The funnel plot of the association between Urea antibody on EoE in MR analysis. UreA, urease subunit-A; EoE, eosinophilic esophagitis.
